# A scoping review of published reviews on the use of bracing in scoliosis

**DOI:** 10.1007/s43390-026-01319-9

**Published:** 2026-03-03

**Authors:** Danielle Hahn, Amy Artajos, Xanthe Elliot, Tara Jevric, Abigail Moore, Lizzie Swaby, Dan Hind, Ashley A. Cole

**Affiliations:** 1 SCHARR, University of Sheffield, Sheffield, S1 4DA UK; 2 School of Healthcare, University of Leeds, Leeds, UK; 3 Department of Paediatric Orthopaedics, Sheffield Children’s Hospital, Sheffield, UK

**Keywords:** Scoliosis, Bracing, Scoping review

## Abstract

**Purpose:**

Scoliosis is a three-dimensional spinal deformity exceeding 10 degrees. Left untreated, it can lead to comorbidities, as well as surface deformity. Brace treatment is common in smaller curves (20–40 degrees), with strong evidence in adolescent idiopathic scoliosis (AIS), but overall effectiveness and impact on quality of life is controversial. This scoping review sought to map existing reviews on all aspects of bracing in scoliosis of any aetiology, to identify future research priorities.

**Methods:**

Searches were conducted in MEDLINE and EMBASE, excluding abstracts, narrative reviews and guidelines. Included articles reported on scoliosis patients of any age and aetiology and examined the use of spinal bracing. Four independent reviewers screened articles for inclusion and completed data extraction. Data were summarised narratively in themes, looking at effectiveness of bracing in AIS, patient factors influencing outcomes of AIS brace treatment, interventions to improve bracing success in AIS, patient and family experiences with bracing in AIS and bracing in adult and neuromuscular scoliosis. AMSTAR2 was used to assess confidence in the results in the reviews.

**Results:**

Searches yielded 59 eligible studies which were included. Whilst bracing is recommended for curves 20°–40° in AIS, it may be successful in those over 40° with good compliance. Bracing is effective in lowering rates of curve progression in AIS and therefore reducing surgery rates. There is no strong evidence that one brace type produces superior outcomes over another, compared to other treatments. Brace adherence is associated with significantly lower rates of curve progression; this is affected by appearance, comfort and psychology. Evidence shows adherence improves with sensor monitoring and psychosocial interventions. Some evidence suggests in-brace correction can be predicted by curve flexibility. More remaining growth potential and associated factors (younger age, lower Risser stage, pre-menarchial, open triradiate cartilage) can increase the risk of curve progression during bracing. Scoliosis-specific exercises may be beneficial alongside brace treatment. Long-term QoL does not appear to be affected by brace treatment. Some low-quality evidence suggests reduced QoL during bracing compared to observation. Function may be impacted by brace treatment, but pain is not increased. In degenerative spinal deformity, there may be some shorter term benefit for pain and function. Little evidence on bracing in neuromuscular scoliosis exists.

**Conclusion:**

There is a large research base of evidence to support bracing for AIS; however, this base is limited due to the substantial amount of low-quality research it includes. The aim of this scoping review was to identify gaps in the literature to guide future research.

This comprehensive review captured the breadth of existing review evidence on all aspects of bracing in scoliosis. Evidence supports bracing as an effective treatment in scoliosis, controlling curve progression, and often increasing patient satisfaction. Compliance is key, and measures such as compliance sensors can be effective. This scoping review has summarised the existing literature; however, the evidence base is limited. Further research could explore objective measures for compliance monitoring, optimal treatment protocols around brace cessation and effects of bracing on patient quality of life.

## Background

Scoliosis is a three-dimensional deformity of the spine where lateral curvatures exceed 10 degrees [1, 2]. Scoliosis is most commonly diagnosed in adolescence, and if left untreated, the curvature can progress and cause a surface deformity alongside an associated increased risk of health problems [3]. Bracing is a common non-surgical treatment. However, its effectiveness and impact on quality of life remain controversial [4]. The effectiveness of bracing has strong evidence in adolescent idiopathic scoliosis (AIS), in patients with curves 20–40 degrees with skeletal maturity of Risser 0–2, but not in other causes of scoliosis [5]. Many reviews of bracing in scoliosis have been published in the last twenty years, synthesising studies on different aspects of bracing for AIS and adult scoliosis. These studies cover a broad range of topics such as success of bracing and factors affecting success, combining bracing with other treatments, bracing compliance, the impact of varying interventions on compliance, effect of different brace types and quality of life. [6–12]. In 2014, an overview of systematic reviews on non-surgical interventions in AIS found most of the reviews to be of low methodological quality, and the higher quality reviews provided insufficient evidence to guide on the effectiveness of non-surgical treatments in AIS.

### Purpose of this review

Scoping reviews seek to systematically identify and map the breadth of the available evidence, to investigate in a more exploratory way, what is known about a specific topic. Given that the existing evidence base is difficult to interpret due to variation in brace types, populations studied and outcomes measured, a scoping review methodology was deemed most appropriate for this paper, compared to systematic reviews which require a very clear, focussed research question. This scoping review sought to comprehensively map the nature and extent of existing research into the effectiveness of bracing for managing scoliosis of any aetiology and associated patient outcomes and experiences in any population and setting. Topics commonly studied will usually result in a scoping or systematic review. This study aims to identify knowledge gaps and establish research priorities rather than identifying prescriptive guidelines.

## Method

The review followed the protocol previously published [13]. The Arksey and O’Malley’s six-stage framework was used and reported within the *PRISMA-ScR* guidelines [14].

### Eligibility criteria

Articles were eligible for inclusion in this scoping and systematic review if they reported on scoliosis patients of any aetiology and age, and which examined the use of spinal bracing or orthoses for conservative treatment. All brace types and designs, wear schedules and durations across any healthcare setting in any geographic location were included. Articles where English translations were not publicly available were excluded. Primary experimental and observation studies were excluded as were narrative reviews, abstracts, case studies and guidelines.

### Information sources and search

The search identified reviews using MEDLINE and EMBASE via the Ovid platform, from data inception to the date of searching (23rd September 2024). The search strategies were drafted by the review supervisor and further refined by discussion with the second review supervisor. The Medline search strategy was as follows:

1. exp scoliosis/
2. scoliosis.mp.
3. spinal curvature.mp.
4. curved spine.mp.
5. lateral curvature.mp.
6. kyphoscoliosis.mp.
7. dextroscoliosis.mp.
8. levoscoliosis.mp.
9. vertebral deformity.mp.
10. spinal deformity.mp.
11. spinal malalignment.mp.
12. spinal asymmetry.mp.
13. spinal lateral deviation.mp.
14. trunk asymmetry.mp.
15. or/1–14
16. "Orthotic Devices"/
17. "Braces"/
18. brac*.ti,ab.
19. ortho*.ti,ab.
20. or/16–19
21. (((systematic or state-of-the-art or scoping or literature or umbrella or mapping) adj (review* or overview* or assessment* or map)) or “evidence synthesis” or “review* of reviews” or meta-analy* or metaanaly* or ((systematic or evidence) adj1 assess*) or "research evidence" or metasynthe* or meta-synthe*).ti,ab.
22. systematic review/ or “systematic review (topic)”/ or meta analysis/ or “meta analysis (topic)”/
23. or/21–22
24. 15 and 20 and 23

The Embase search strategy was similar and can be found in the review protocol [13]. The search results were exported to Rayyan, and duplicates were removed by four reviewers.

### Selection of sources of evidence

**Fig. 1:**
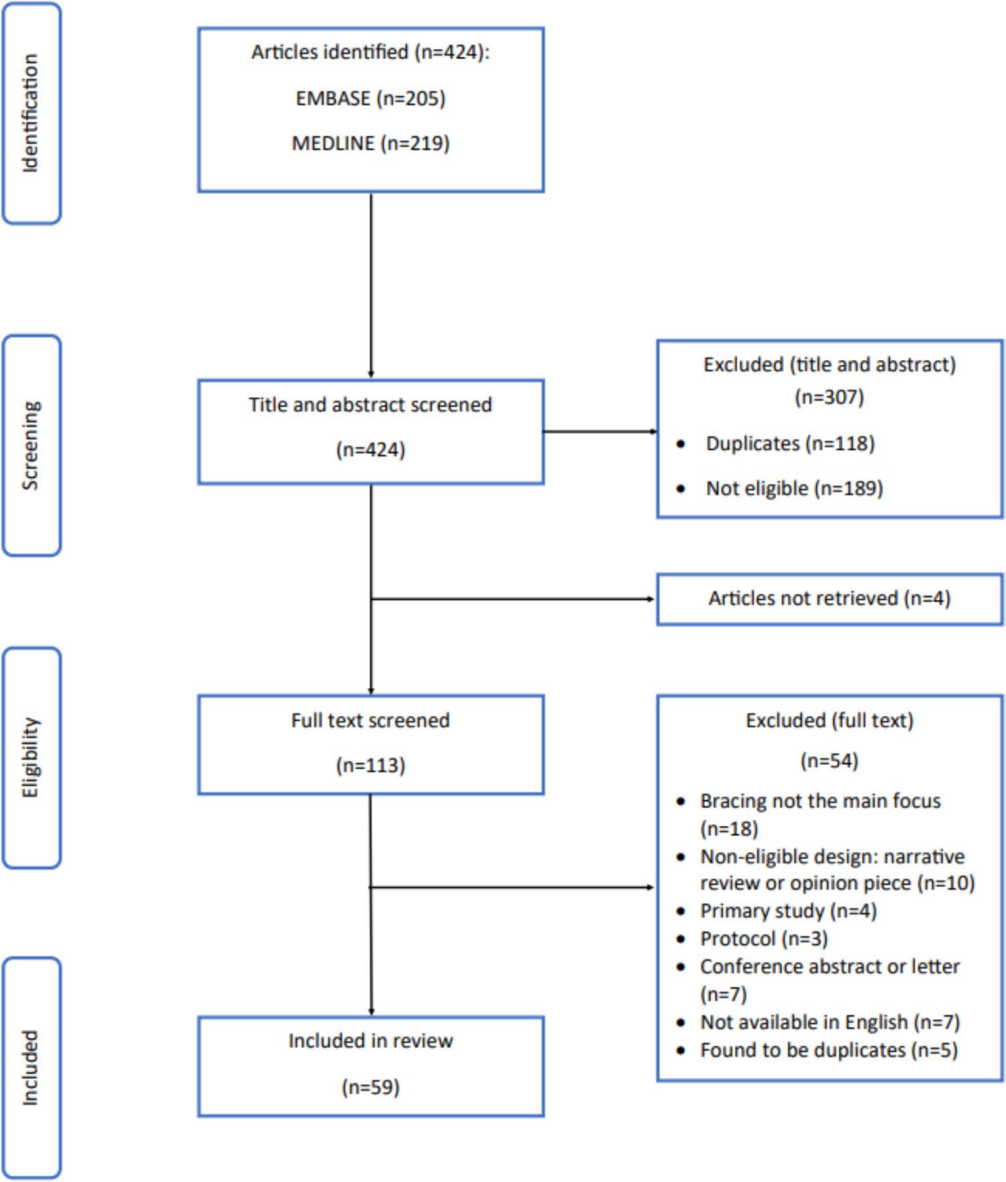
PRISMA-ScR flowchart

Four reviewers independently screened the titles and abstracts against the eligibility criteria; following this the full texts were screened against the same criteria, by the same reviewers. Discrepancies at each review stage were resolved through group discussion, and reasons for full-text exclusion were recorded. The selection process was reported in a PRISMA-ScR flow diagram (Fig. 1).

### Data charting process

A standardised form was developed between reviewers and used to chart the data extracted from each review, which was modified iteratively. Four reviewers independently extracted the data with discrepancies resolved through discussion. Methods of included studies, study designs, sample sizes, setting, brace types and populations, outcome measures and time points were extracted. When charting key findings, authors’ conclusions were recorded. For the charting of research gaps, authors conclusions and uncertainties, alongside recommendations for future research and questions were recorded. Articles were grouped in accordance with the information they provided on the following five objectives and corresponding sub-categories:

- *Effectiveness of bracing in Adolescent Idiopathic Scoliosis (AIS): curve progression; reaching the threshold for surgery; comparison of effectiveness by brace types; bracing compared to other treatments*
- *Patient factors influencing outcomes of AIS brace treatment: brace wear adherence; curve type and magnitude; skeletal maturity, age, BMI, sex.*
- *Interventions to improve success of brace treatment in AIS: sensor monitoring for adherence; exercise and physical therapy; other improvements.*
- *Patient and family experiences with bracing in AIS: quality of life (QoL); psychological and psychosocial impacts; pain and functional outcomes; adverse events*
- *Bracing in adult and neuromuscular scoliosis: effectiveness; indications for brace treatment; complications and adverse effects*

### Synthesis of results

Results were summarised descriptively and graphically in accordance with the five objectives. Summarising was assisted using ‘Notebook LM’, by generating a listed synthesis of key findings and research recommendations. AMSTAR2 was used to assess confidence in the results of the reviews.

## Results

### Selection of sources of evidence

From the database searches, 424 records (Fig. 1) were identified from Embase (*n* = 205) and MEDLINE (*n* = 219). From the initial review of the title and abstract, 307 were excluded, including 118 duplicates, and 117 full-text articles were included. Four articles could not be retrieved, leaving 113 articles screened at full text. A further 54 studies were excluded from the review where bracing was not the main focus (*n* = 18), or non-eligible design: narrative review or opinion piece (*n* = 10), primary study (*n* = 4), protocol (*n* = 3), conference abstract or letter (*n* = 7). Some articles were not available in English (*n* = 7) and others were found to be duplicates (*n* = 5). The remaining 59 studies were considered eligible for this review.

### Characteristics of sources of evidence

The range of publication dates of the included studies was from 1997 [14] to 2024[15–20]. There were 7 studies before 2010, 20 from 2010 to 2019 and 32 from 2020 to 2024. The types of studies included were systematic reviews (*n* = 37) [3, 7, 8, 15, 18–50]; scoping reviews (*n* = 1) [16] and other reviews with systematic methods (*n* = 21) [11, 12, 17, 40, 51–67]. The median number of primary research studies was 12.5 (0 [32] – 60 [64]). Most reviews analysed a range of brace types, with three brace types being significantly more common; these were the Cheneau brace, the Boston brace and the SpineCor brace.

### Synthesis of results

**Fig. 2:**
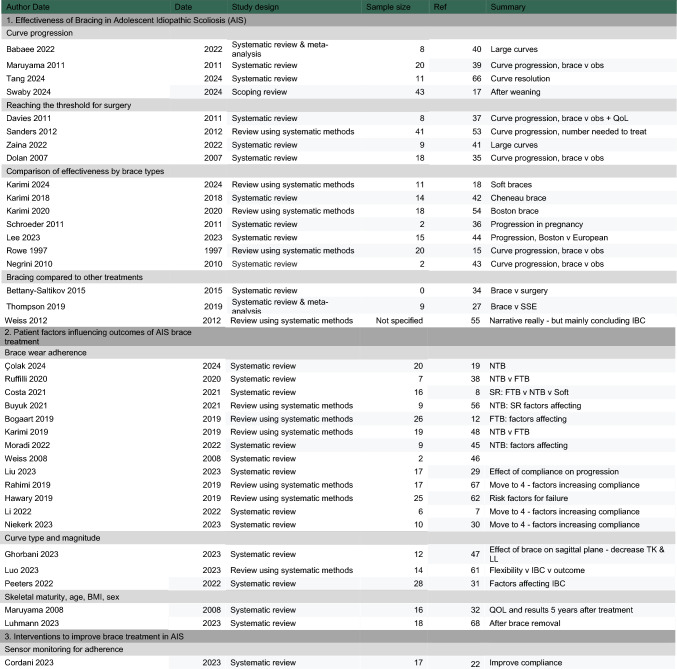

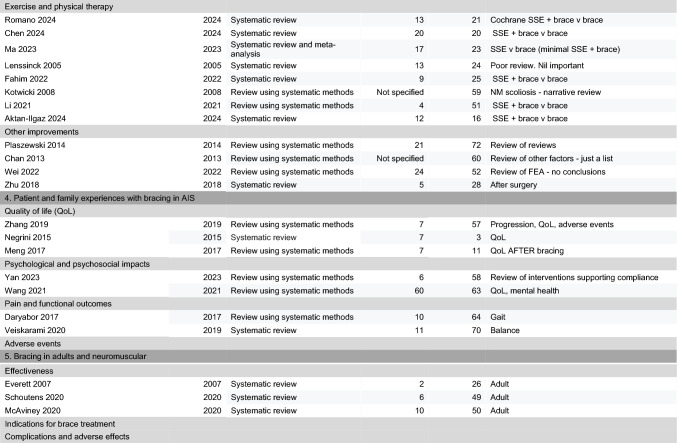
Data Charting table

The reviews were sorted into themes of Effectiveness of bracing in Adolescent Idiopathic Scoliosis (AIS) (*n* = 18); Patient factors influencing outcomes of AIS brace treatment (*n* = 18); Interventions to improve success of brace treatment in AIS (*n* = 13); Patient and family experiences with bracing in AIS (*n* = 7) and Bracing in adults and neuromuscular scoliosis (*n* = 3). Full data extraction tables can be found in Appendix 1 (Fig. 2).

### Effectiveness of bracing in adolescent idiopathic scoliosis (AIS)

The reviews were categorised into subthemes: curve progression (*n* = 4); reaching the threshold for surgery (*n* = 4); comparison of effectiveness by brace types (*n* = 7); bracing compared to other treatments (*n* = 3).

#### Effectiveness of bracing in AIS: curve progression

Curve progression or regression is generally considered as a change in Cobb angle of 6 degrees or more [68]. Bracing is effective in controlling curve progression in AIS, particularly for curves between 25° and 40° [37, 42, 49].

There is some evidence that bracing may be effective for curves larger than 40° with some curves improving, especially with good brace compliance and when combined with scoliosis-specific exercises for curves 40°–60° [38, 39, 49].

Bracing cessation and weaning protocols varied across literature with evidence of curve progression in many patients after brace cessation [16, 50]. Curve progression related to pregnancy may be more common in previously braced patients [34].

#### Effectiveness of bracing in AIS: reaching the threshold for surgery

Studies have reported surgery rates, but it is more common to use curve progression to 50 degrees or more as a surrogate marker of surgery. Two older reviews from 2007 [33] and 2011 [35] found no evidence for bracing reducing the number of patients reaching a surgical threshold, whilst reviews from 2010 [41] and 2011 [37] did suggest that bracing reduces the risk of curve progression. More recent reviews have concluded that bracing is effective in reducing the number of AIS patients reaching a surgical threshold [3, 8, 40, 42, 54, 58]. The number needed to treat (NNT) for each surgery prevented is approximately nine patients in routine scoliosis bracing [54]. There is limited evidence to suggest bracing may be effective in preventing surgery for highly compliant patients motivated to avoid surgery, with the NNT reducing to 4 in such instances. However, limitations of these findings are acknowledged [39, 54].

#### Effectiveness of bracing in AIS: comparison of effectiveness by brace types

The evidence suggests SpineCor brace (flexible brace) is effective for controlling curves under 30° in patients with Risser stages 0–3 [17]. The SpineCor brace is effective in achieving curve stabilisation with high compliance [17] but probably has lower success than a rigid brace [8].

The Boston, Chêneau and other ‘European’ braces are effective in controlling scoliotic curve progression of the lumbar and thoracolumbar region in particular [40, 42, 55]. There is no conclusive evidence that one FTB is better than another [42].

The Milwaukee brace demonstrated high effectiveness in halting curve progression when worn for 23 h per day but is no longer used [51].

One review compared AIS patients treated in full-time brace (FTB) with night-time only bracing (NTB) finding no difference between the two braces in 5 studies and superiority of FTB in 2 studies and so concluded that there is insufficient evidence to draw a conclusion about NTBs [36]. Two meta-analyses evaluated curve progression in AIS patients treated with NTB finding no significant difference to FTB [8, 57]. Two further reviews of NTB with narrative analyses did not draw a conclusion and suggested a randomised controlled trial [18, 46].

#### Effectiveness of bracing in AIS: bracing compared to other treatments

When compared to other conservative treatments, bracing seems to be more effective than electrical stimulation [37]. There are three reviews evaluating bracing against scoliosis-specific exercises. They were inconclusive regarding the effect on the curve, mental health may be better in the SSE group and no difference was found in quality of life measures [20, 22, 26]. A previous Cochrane review found no studies comparing surgery with bracing [32]. Older reviews found insufficient evidence [23].

### Patient factors influencing outcomes of AIS brace treatment

The reviews were sorted into subthemes with many papers considering multiple factors: Brace wear adherence (*n* = 13); Curve type and magnitude (*n* = 3); Skeletal maturity, age, BMI, sex (*n* = 2).

#### Patient factors influencing outcomes of AIS brace treatment: brace wear adherence

There are four reviews evaluating the importance of brace adherence, which have also considered the quality of the evidence and all agree that brace adherence is associated with significantly lower curve progression and lower rates of progression to a surgical threshold [12, 28, 44, 63]. Similarly, lower adherence is associated with higher failure. Optimal adherence has not been defined.

Brace adherence is affected by brace appearance, comfort, prescription (FTB or NTB) and psychological factors such as involving the patient and family in the treatment plan and the use of temperature sensors to objectively monitor adherence [66].

#### Patient factors influencing outcomes of brace treatment: curve type and magnitude

There is moderate to strong evidence that in-brace correction is associated with FTB treatment success (if high) and failure (if low) [12, 38, 49, 56, 62, 63]. There is moderate evidence that in-brace correction is predicted by curve flexibility usually assessed on supine radiographs [30, 62] and that this is also associated with increased NTB success [43].

There is weak or conflicting evidence on the success of full-time and night-time only bracing for curve type, curve size (in the bracing range of 20–40 degrees), apical vertebral rotation, translation and wedging, curve apex, rib-vertebral angle, coronal imbalance, angle of thoracic rotation, osteopenia, plane of maximal curvature and hypokyphosis [12, 38, 43, 49, 57, 63].

#### Patient factors influencing outcomes of brace treatment: skeletal maturity, age, BMI, sex

For FTB, there is some evidence that factors associated with more remaining growth potential at the onset of bracing (younger age, lower Risser stage, pre-menarchial, open triradiate cartilage) are associated with an increased risk of curve progression during bracing [12, 38, 63]. There is limited evidence regarding bracing success and failure for BMI (high and low) and sex [12, 63].

For NTB, there is limited evidence that a more immature Risser stage may be associated with an increased risk of curve progression and that a more mature Risser stage may be associated with a reduced risk of curve progression [43, 57].

### Interventions to improve success of brace treatment in AIS

The included reviews were sorted into following subthemes: Sensor monitoring for adherence (*n* = 1); Exercise and physical therapy (*n* = 8); Other improvements (*n* = 4).

#### Interventions to improve brace treatment in AIS: sensor monitoring for adherence

There is strong evidence to show that when AIS patients are aware that their adherence is being monitored, brace adherence improves by 3.5 h/day [7, 21, 29]. Most studies have used temperature sensors but others have used pressure sensors to measure adherence.

Psychosocial interventions such as cognitive behavioural therapy (CBT) also improves compliance by approximately 3 h/day [7, 21, 29, 59, 66]. Combining sensor monitoring with CBT seems to have a cumulative effect [21]. There is limited evidence on the effects of scoliosis-specific exercises and education on brace adherence. Improving the brace appearance, brace comfort and involving the patient and family more in the treatment plan may improve brace adherence [7, 66].

#### Interventions to improve brace treatment in AIS: exercise and physical therapy

There is moderate or low-certainty evidence that compared with brace treatment, a combination of bracing and scoliosis-specific exercises (SSE, including Schroth exercises) is significantly better for Cobb angle [15, 19, 20, 24, 52]. There is conflicting and limited evidence that combining bracing and SSE may improve subjective appearance, self-image, quality of life and pulmonary function [15, 19, 20].

#### Interventions to improve brace treatment in AIS: other improvements

A review of other potential improvements to brace treatment includes lower radiation dose treatment monitoring (EOS imaging, ultrasound); brace design (CAD/CAM, finite element models); in-brace pressure monitoring to consider ‘quality’ of brace wear and better questionnaire assessment of quality of life [53, 61, 67, 73]. No conclusions were reached. A review of postoperative bracing after spine surgery included only one scoliosis study and shows the lack of evidence in this area [27].

### Patient and family experiences with bracing in AIS

The reviews were categorised into subthemes: Quality of life (*n* = 3); Psychological and psychosocial impacts (*n* = 2); pain and functional outcomes (*n* = 2); Adverse events.

#### Patient and family experiences with bracing in AIS: quality of life (QoL)

The studies within the reviews mostly use SRS-22 and PEDQL questionnaires to assess QoL. The reviews compare brace treated to observed patients with one review finding QoL better in braced patients [58] and three finding no significant difference in QoL between braced and observed patients [3, 11, 35]. There is limited evidence suggesting that long-term QoL, back pain, psychological and cosmetic issues are not affected by brace treatment [3, 31].

#### Patient and family experiences with bracing in AIS: psychological and psychosocial impacts

There is low-quality evidence that bracing negatively affects self-image, mental health and psychological stress [64]. Once brace treatment is complete, there is probably no difference between previous brace-treated patients and previously observed patients for self-image and mental health [11].

Psychosocial interventions have been shown to improve brace compliance (see above) but there is no evidence to suggest improvements in self-esteem, social adjustment and mental health in brace-treated patients [59].

#### Patient and family experiences with bracing in AIS: pain and functional outcomes

During bracing, there is surprisingly very little evidence that bracing causes increased pain; however, more evidence that function and vitality are adversely affected including gait and balance [64, 65, 69]. Once brace treatment is complete, there is no evidence that pain and function are any different from patients treated with observation [11].

#### Patient and family experiences with bracing in AIS: adverse events

There is conflicting evidence regarding adverse events with a systematic review finding them more common in the braced patients [58] and a Cochrane review finding no significant difference between brace and observed patients [3].

### Bracing in adults and neuromuscular scoliosis

All three reviews on brace treatment in adult/degenerative spinal deformity agree that there is insufficient evidence, although there may be some short- to medium-term benefit for pain and function [24, 46, 47].

There is very little evidence on bracing in neuromuscular scoliosis. Bracing may be more difficult in neuromuscular scoliosis and less well tolerated with no current evidence for reducing the risk of curve progression or other benefits [60].

## Discussion

### Summary of findings

We created a map of evidence that included 59 reviews addressing bracing for scoliosis across predominantly adolescent populations published between 1997 and 2024. Most of the research has been done in patients with AIS. Quality of the review evidence is not typically done for scoping reviews but AMSTAR 2 assessment to assess the confidence in the results was performed for the 31 reviews in the discussion: 1 was assessed as ‘moderate’ [49]; 9 as ‘low [7, 12, 15, 19, 28, 29, 42, 59, 62] and 21 as critically low [8, 11, 18, 20, 21, 24, 30, 36, 38, 39, 43–46, 52, 56, 57, 61, 63, 64, 66]. AMSTAR is a ‘high bar’ with reviews assessed as ‘moderate’ and ‘low’ probably being of good quality for bracing reviews. Our findings indicate that bracing in AIS can be effective at preventing curve progression and reaching the threshold for surgical treatment in scoliosis [ref]. There is good evidence that this is largely determined by brace adherence [12, 28, 44, 63] and in-brace correction [12, 38, 56, 61–63]. Other potential baseline factors such as curve type, Cobb angle, apical vertebral rotation, apical wedging, rib-vertebral angle, coronal plane imbalance, angle of thoracic rotation, osteopenia, plane of maximal curvature and hypokyphosis require more evidence [30, 43, 49, 56, 62]. Bracing in AIS does produce curve regression in some patients.

Whilst bracing is recommended for curves 20°–40° in AIS patients with growth potential, it may be successful in those with curves over 40° [38, 39, 49] with the review by Tang et al. 2024 [49] assessed as AMSTAR 2 ‘moderate’ confidence. There is no strong evidence that one brace type produces superior outcomes over another due to the wide range of results and absence of a meta-analysis [42]. There is limited evidence that night-time only bracing may be as effective as full-time bracing, although which curves are suitable is not established [8, 18, 36, 43, 46, 57]. Whilst there is no evidence that bracing has an adverse effect on QoL [3, 11, 35, 58], there may be a negative effect on mental health, self-image and psychological stress during bracing which seems to resolve once bracing is finished [11, 64]. The reviews do not all agree representing the low quality of evidence on this important issue.

Some of the better quality reviews suggest psychosocial interventions and brace wear-time monitoring probably improve adherence which has a positive effect on bracing success [7, 21, 29, 59, 66]. Again there is evidence from some of the better quality reviews that brace success can be improved by the addition of SSE (including Schroth exercises) [15, 19, 20, 24, 52].

There was only one review mentioning bracing in neuromuscular scoliosis and no reviews on bracing in early onset or congenital scoliosis, although the authors are aware of a review on bracing in early onset scoliosis published after the searches were done for this review [70]. Evidence of bracing in patients with a diagnosis other than AIS is very limited, and there are no reviews of postoperative bracing.

Each review was evaluated for suggestions for future research, and these are summarised in Table 1. It is not practical to evaluate many of the research questions using RCTs due to lack of clinician and patient equipoise in treatment options which would limit recruitment and make the trial too long and too expensive. If it can be firmly established that an RCT is not possible, then a pragmatic observational study methodologically minimising bias and confounding can help resolve the issues. One review noted: “The potential for consistently increasing the strength of evidence is in question, in a field where parents reject randomisation of their children, as demonstrated by the high rate of failure of RCTs” [3].

**Table 1:** Suggestions for future bracing research from the reviews (research suggestions from early reviews which have been performed and included in subsequent reviews will not be included)

Category	Identified gaps and recommendations
Study design	● Adherence to Scoliosis Research Society (SRS) and Society on Scoliosis Orthopaedic and Rehabilitation Treatment (SOSORT) guidance for patient selection and reporting of short and long-term outcomes [41, 45, 57] ● For stratification or assessment of confounding variables, baseline parameters such as age, sex, deprivation indices, Risser sign, Cobb angle, curve type, gender, treatment compliance and progression factor estimation should be determined [8, 18, 38, 54, 57, 59] ● Assessment of skeletal maturity with Sanders’ staging and other skeletal maturity indicators to improve clinical relevance of each study and allow for direct and accurate comparisons between studies [8, 38, 54, 57] ● Reporting of objective measures of wear time based on monitors within the braces [7, 21, 38, 49, 57]. Evaluate the possible confounding factors of environmental temperature and brace design [7] ● Outcome measures to include: ○ Sagittal parameters [45] ○ Quality of Life, self-image, mental health, pain, function, vitality and satisfaction [11, 19, 20, 22, 64] ○ Cost effectiveness of bracing [26, 33, 35] ● Rigorous methods and reporting standards [26] ● Long-term follow-up [19, 20, 22] ● Bracing studies should evaluate adverse events [41, 58]
Effectiveness of bracing	*Curve progression and reaching the threshold for surgery* ● Evaluation of factors for curve progression during brace treatment [16, 40, 50] ● Evaluation of factors responsible for curve regression during brace treatment [49] ● Evaluation of the factors that predict the outcome of brace treatment for AIS patients with ≥ 40° curves [38, 39] ● Clearer and more consistent guidelines for comparison between bracing and surgery (larger curves only) [37] *Skeletal Maturity, brace stopping and weaning* ● Evaluate the best skeletal maturity measures [8, 54] ● Any benefit to weaning and the best time to stop bracing based on risk [16] ● A better understanding of curve progression risk after brace removal, including curve type and size, may allow an estimate of future prognosis, [16, 46, 66] *Long-term follow-up* ● Long-term studies to determine whether ‘patient-determined’ rate of surgery is more appropriate than the more typical ‘clinically determined’ rate of surgery in terms of health and function throughout adulthood [33] ● The interaction of pregnancy and curve progression [34] *Comparison of effectiveness by brace types* ● Evaluate the effectiveness of different full-time brace types [17, 40] ● RCT’s comparing effectiveness of night-time bracing (NTB) with full-time bracing with a large sample over a long follow-up period [40] ● Evaluation of factors affecting the success and failure of different brace types including NTB [43]
Factors influencing brace treatment outcomes	*Brace wear adherence* ● Effect of brace adherence on success and failure of different brace types and regimes [12, 28] ● Effect of prescribed wear time on patient compliance [51] ● Evaluate the true effect of bracing (efficacy) in highly compliant patients who are compliant with SSE [42, 49] ● Evaluation of the effect on brace compliance of starting brace wear at an earlier age and considering child habits and preferences in the treatment plan and the prescribed regimen [66] *In-brace correction* ● Provide both absolute and percentage curve corrections when identifying potential predictive factors on initial in-brace correction [12, 30] ● Future studies should explore strategies to improve spinal flexibility before bracing [62]
Interventions to improve brace treatment	*Sensor monitoring for adherence* ● To confirm the actual benefit of compliance-improving interventions in clinical practice [7] *Exercise and physical therapy* ● RCT’s for the value of scoliosis-specific exercises being added to bracing and whether there is any difference between physiotherapeutic scoliosis-specific exercises and generic therapeutic exercises [19, 20, 22] ● Standardise intervention protocols as validity of findings is constrained by variability of exercise protocols and inadequate methodological quality [22, 24] ● Provide comprehensive explanations about compliance to exercises and braces and the sorts of braces and exercises used [9] *Other improvements* ● Development and testing of psychosocial interventions for paediatric patients with emphasis on multidisciplinary teams delivering holistic care [29, 42] ● RCT to confirm whether postoperative bracing improves outcomes after spinal surgery [27]
Patient and family experiences	*Functional outcomes* ● The effect of bracing on gait in AIS; comparison between the rigid and soft orthoses on gait parameters and energy consumption; evaluation of the whether excessive energy cost of walking is due to poor physical condition or muscular disease; evaluate the cause of the excessive energy consumption in braced AIS patients; EMG when walking in AIS patients with and without brace [65] ● The effect of bracing on balance parameters in subjects with AIS for an appropriately longer period; comparison between rigid and dynamic scoliosis orthoses on balance parameters and energy consumption; balance parameters in scoliotic subjects following spinal surgery [55] ● The effect of bracing on pulmonary disorders and disability [3]
Bracing in other types of scoliosis	*Adult and degenerative scoliosis* ● To better understand the influence of bracing in adult scoliosis, prospective cohort studies should [48]: o Include an adequate and representative sample of both cases and matched controls; o Have clearly defined diagnoses o Utilise standardised patient-centred clinical and radiographic outcomes; and ● Establish the effectiveness of the use of bracing, yoga and injections and other non-surgical treatments [47] ● The use of custom or general “off the shelf” thoracolumbar orthosis, wearing patterns and progression with brace treatment compared to observation [25] ● The frequency and duration of physical therapy and chiropractic care needed to establish a clinical effect and the impact of bracing on core strength [25, 47] *Neuromuscular scoliosis* ● There are no suggestions for future bracing research in neuromuscular scoliosis *Early Onset Scoliosis* ● There are no suggestions for future bracing research in early onset scoliosis *Congenital Scoliosis* ● There are no suggestions for future bracing research in congenital scoliosis

### Scoliosis brace treatment randomised controlled trials (RCT)

A scoping review like this aiming to identify research gaps in brace treatment for scoliosis must acknowledge currently available high-quality research (Randomised Controlled Trials). Weinstein et al. (2013) in a part randomised, part preference trial showed that 72% of the brace-treated AIS patients attained skeletal maturity with a curve less than 50 degrees compared to 48% in the observation group. Sub-group analysis suggested a strong relationship between brace compliance and success with those braced for less than 6 h per day achieving the same success rate as the observation cohort and 80% achieving success if bracing more than 12.9 h per day. Miller et al. (2012) in a small RCT of 21 patients found patients who were told they had a monitor in their brace to assess compliance had 5.24 h more compliance than those uninformed over 14 weeks. Cheung et al. (2024) randomised 369 AIS patients to immediate brace cessation or gradual weaning (night-time only wearing for 6 months). The immediate weaning group had mean 3.2 degrees of curve progression at 2 years after skeletal maturity compared to 3.0 degrees in the gradual weaning group with no significant difference between the groups. Guo et al. (2014) randomised 38 AIS patients to either a rigid full-time brace or a soft brace (SpineCor), finding higher curve progression in the SpineCor group. Charalampidis et al. (2023) performed an RCT (CONTRAIS study) comparing night-time bracing with scoliosis-specific exercises (SSE) and physical activity alone (control). They had 45 patients in each arm and found night-time bracing reduced the number of patients progressing by 6 degrees or more over 2 years compared with physical activity whilst SSE did not. Peiro-Garcia et al. (2025) in an RCT of night-time versus full-time bracing in 78 AIS patients found better self-image and reduced pain in the night-time braced patients by 12 months. Lin et al. (2021) found no difference in conventionally manufactured full-time braces versus 3D printed braces in the 22 AIS patients after 2 years. Zheng et al. (2018) randomised 60 AIS patients to full-time bracing or Scientific Exercise Approach to Scoliosis. Of the 53 patients analysed at 12 months, bracing improved Cobb angle and aesthetics more than exercises but the exercise patients favoured better in terms of function and psychological status. Corbetto et al. (2016) randomised 40 AIS patients to standard CAD/CAM design or design with the addition of information from a finite element model (FEM) of the trunk and spine allowing simulation of brace correction. Braces designed with the addition of FEM had better in-brace correction of the thoracic curve and were lighter.

### Strengths of findings

This review was a broad, comprehensive search across multiple databases to capture the breadth of the existing evidence from review articles on bracing for scoliosis. The eligibility criteria were highly inclusive, spanning a wide range of scoliosis aetiologies, age groups, brace types and outcomes to generate a high-quality map of existing evidence. The review followed an explicit, pre-specified protocol depicting an established scoping review framework of PRISMA-ScR [13]. The risk of bias and errors was reduced by four reviewers independently conducting screening and data charting. An experienced paediatric scoliosis surgeon (AAC) reviewed all the papers, data extraction and suggestions for further research.

### Limitations of findings

This was a scoping review and therefore did not include a quality appraisal of included reviews. Scoping review methodology does not generate conclusive answers or practice recommendations [71]. This means that findings are limited by the quality of included reviews, with most reviews concluding that there is a need for more high-quality research into bracing in scoliosis, especially when conducting long-term prospective studies. Infrequently studied topics related to brace treatment are unlikely to be subjected to a scoping or systematic review and will therefore not be identified in this paper. This is the case for bracing in adult and neuromuscular scoliosis and postoperative bracing.

Due to the exclusion criteria, relevant studies may have been omitted that were not published in the English language, and studies not indexed in the selected databases may have been missed.

Although data charting was conducted by four reviewers, a single reviewer extracted from each review, which could have introduced inconsistencies in charted content. Certain studies that were captured by multiple reviews may be overemphasised.

Four eligible studies were irretrievable. It was concluded from the title and abstract of these reviews that they were unlikely to add to the evidence presented in this review.

### Further research

There is a consistent theme recommending a need for research that follows the Scoliosis Research Society (SRS) guidelines for bracing in AIS [72] and which are of a higher quality, for more effective evaluation of brace treatment [35, 38, 39, 54]. There is a need for the SRS guidelines on bracing to be reviewed as currently bracing is recommended for curves 25°–40°, whilst the RCT proving the effectiveness of bracing in AIS was for 20°–40° curves [5].

Many reviews advocate for assessing patient-centred outcomes at short, medium and long-term follow-up points [41, 45, 48], in addition to measuring Cobb angles over time [41, 45], alongside consideration of factors which may affect success and complimentary treatments that may improve outcomes.

There is a lack of research covering long-term follow-up periods in bracing generally, which limits findings pertaining to its effectiveness overall [18, 19, 33, 40, 41, 45, 48, 58].

Future research could address the implementation of objective measures for compliance monitoring into clinical practice, such as temperature sensors [21]; the optimal treatment protocols including any benefits from psychological input and scoliosis-specific exercises, concerning weaning time and cessation [16, 50]; the effects of bracing on patient quality of life and function [3, 11, 58].

### Implications for treatment

There is a need for braced patients' mental health and quality of life to be a priority when devising treatment plans, reviews have agreed that compliance is heavily influenced by these two aspects [29, 45, 66]. Focus to improve compliance should be through sensor monitoring and an integrated clinical team approaches [29, 45, 66]. Combining bracing with SSE may be effective treatment for mild to moderate AIS [19, 52].

## Conclusions

There is a large research base of evidence to support bracing for AIS; however, this base is limited due to the substantial amount of low-quality research it includes. The aim of this scoping review was to identify gaps in the literature to guide future research.

Evidence supports bracing in AIS as effective in reducing the risk of curve progression and reaching the surgical threshold. Bracing sometimes results in a reduction of the curve and may be useful in curves over 40°. In AIS patients, bracing compliance and in-brace correction seem to be the best predictors of bracing success. Psychosocial interventions and brace wear-time monitoring probably improve adherence which has a positive effect on bracing success. There is some evidence that brace success can be improved by the addition of SSE.

## Data Availability

Data sharing is not applicable to this article as no datasets were generated or analysed during the current study.

## Appendix 1

**Table:**

Author Date	Date	Study design	Sample size	Ref	Summary
1. Effectiveness of Bracing in Adolescent Idiopathic Scoliosis (AIS)					
Curve progression					
Babaee 2022	2022	Systematic review & meta-analysis	8	40	Large curves
Maruyama 2011	2011	Systematic review	20	39	Curve progression, brace v obs
Tang 2024	2024	Systematic review	11	66	Curve resolution
Swaby 2024	2024	Scoping review	43	17	After weaning
Reaching the threshold for surgery					
Davies 2011	2011	Systematic review	8	37	Curve progression, brace v obs + QoL
Sanders 2012	2012	Review using systematic methods	41	53	Curve progression, number needed to treat
Zaina 2022	2022	Systematic review	9	41	Large curves
Dolan 2007	2007	Systematic review	18	35	Curve progression, brace v obs
Comparison of effectiveness by brace types					
Karimi 2024	2024	Review using systematic methods	11	18	Soft braces
Karimi 2018	2018	Systematic review	14	42	Cheneau brace
Karimi 2020	2020	Review using systematic methods	18	54	Boston brace
Schroeder 2011	2011	Systematic review	2	36	Progression in pregnancy
Lee 2023	2023	Systematic review	15	44	Progression, Boston v European
Rowe 1997	1997	Review using systematic methods	20	15	Curve progression, brace v obs
Negrini 2010	2010	Systematic review	2	43	Curve progression, brace v obs
Bracing compared to other treatments					
Bettany-Saltikov 2015	2015	Systematic review	0	34	Brace v surgery
Thompson 2019	2019	Systematic review & meta-analysis	9	27	Brace v SSE
Weiss 2012	2012	Review using systematic methods	Not specified	55	Narrative really—but mainly concluding IBC
2. Patient factors influencing outcomes of AIS brace treatment					
Brace wear adherence					
Çolak 2024	2024	Systematic review	20	19	NTB
Ruffilli 2020	2020	Systematic review	7	38	NTB v FTB
Costa 2021	2021	Systematic review	16	8	SR: FTB v NTB v Soft
Buyuk 2021	2021	Review using systematic methods	9	56	NTB: SR factors affecting
Bogaart 2019	2019	Review using systematic methods	26	12	FTB: factors affecting
Karimi 2019	2019	Review using systematic methods	19	48	NTB v FTB
Moradi 2022	2022	Systematic review	9	45	NTB: factors affecting
Weiss 2008	2008	Systematic review	2	46	
Liu 2023	2023	Systematic review	17	29	Effect of compliance on progression
Rahimi 2019	2019	Review using systematic methods	17	67	Move to 4—factors increasing compliance
Hawary 2019	2019	Review using systematic methods	25	62	Risk factors for failure
Li 2022	2022	Systematic review	6	7	Move to 4—factors increasing compliance
Niekerk 2023	2023	Systematic review	10	30	Move to 4—factors increasing compliance
Curve type and magnitude					
Ghorbani 2023	2023	Systematic review	12	47	Effect of brace on sagittal plane—decrease TK & LL
Luo 2023	2023	Review using systematic methods	14	61	Flexibility v IBC v outcome
Peeters 2022	2022	Systematic review	28	31	Factors affecting IBC
Skeletal maturity, age, BMI, sex					
Maruyama 2008	2008	Systematic review	16	32	QOL and results 5 years after treatment
Luhmann 2023	2023	Systematic review	18	68	After brace removal
3. Interventions to improve brace treatment in AIS					
Sensor monitoring for adherence					
Cordani 2023	2023	Systematic review	17	22	Improve compliance
Exercise and physical therapy					
Romano 2024	2024	Systematic review	13	21	Cochrane SSE + brace v brace
Chen 2024	2024	Systematic review	20	20	SSE + brace v brace
Ma 2023	2023	Systematic review and meta-analysis	17	23	SSE v brace (minimal SSE + brace)
Lenssinck 2005	2005	Systematic review	13	24	Poor review. Nil important
Fahim 2022	2022	Systematic review	9	25	SSE + brace v brace
Kotwicki 2008	2008	Review using systematic methods	Not specified	59	NM scoliosis—narrative review
Li 2021	2021	Review using systematic methods	4	51	SSE + brace v brace
Aktan-Ilgaz 2024	2024	Systematic review	12	16	SSE + brace v brace
Other improvements					
Plaszewski 2014	2014	Review using systematic methods	21	72	Review of reviews
Chan 2013	2013	Review using systematic methods	Not specified	60	Review of other factors—just a list
Wei 2022	2022	Review using systematic methods	24	52	Review of FEA—no conclusions
Zhu 2018	2018	Systematic review	5	28	After surgery
4. Patient and family experiences with bracing in AIS					
Quality of life (QoL)					
Zhang 2019	2019	Review using systematic methods	7	57	Progression, QoL, adverse events
Negrini 2015	2015	Systematic review	7	3	QoL
Meng 2017	2017	Review using systematic methods	7	11	QoL AFTER bracing
Psychological and psychosocial impacts					
Yan 2023	2023	Review using systematic methods	6	58	Review of interventions supporting compliance
Wang 2021	2021	Review using systematic methods	60	63	QoL, mental health
Pain and functional outcomes					
Daryabor 2017	2017	Review using systematic methods	10	64	Gait
Veiskarami 2020	2019	Systematic review	11	70	Balance
Adverse events					
5. Bracing in adults and neuromuscular					
Effectiveness					
Everett 2007	2007	Systematic review	2	26	Adult
Schoutens 2020	2020	Systematic review	6	49	Adult
McAviney 2020	2020	Systematic review	10	50	Adult
Indications for brace treatment					
Complications and adverse effects					
